# Problems with using mechanisms to solve the problem of extrapolation

**DOI:** 10.1007/s11017-013-9266-0

**Published:** 2013-07-17

**Authors:** Jeremy Howick, Paul Glasziou, Jeffrey K. Aronson

**Affiliations:** 1 Department of Primary Care Health Sciences, Centre for Evidence-Based Medicine, University of Oxford, New Radcliffe House, Radcliffe Observatory Quarter, Woodstock Road, Oxford, OX2 6GG UK; 2 Faculty of Health Sciences and Medicine, Bond University, Gold Coast, QLD 4229 Australia

**Keywords:** Randomized trials, Evidence-based medicine, Mechanism, External validity, Implementation, Extrapolation, Nancy Cartwright

## Abstract

Proponents of evidence-based medicine and some philosophers of science seem to agree that knowledge of mechanisms can help solve the problem of applying results of controlled studies to target populations (‘the problem of extrapolation’). We describe the problem of extrapolation, characterize mechanisms, and outline how mechanistic knowledge might be used to solve the problem. Our main thesis is that there are four often overlooked problems with using mechanistic knowledge to solve the problem of extrapolation. First, our understanding of mechanisms is often (and arguably, likely to remain) incomplete. Secondly, knowledge of mechanisms is not always applicable outside the tightly controlled laboratory conditions in which it is gained. Thirdly, mechanisms can behave paradoxically. Fourthly, as Daniel Steel points out, using mechanistic knowledge faces the problem of the ‘extrapolator’s circle’. At the same time, when the problems with mechanistic knowledge have been addressed, such knowledge can and should be used to mitigate (nothing can entirely solve) the problem of extrapolation.

## Introduction

Philosophers of science have recently argued that studying mechanisms is useful for addressing many conundrums in science and the philosophy of science. A paper that sparked recent interest in mechanisms concluded: ‘if one does not think about mechanisms, one cannot understand neurobiology and molecular biology’ [1, p. 24]. Investigating mechanisms also allegedly helps provide an account of causation [2–4], scientific explanation [4, 5], and Glennan even argues that providing a mechanism solves Hume’s problem of induction [2]. Philosophical work on mechanisms has expanded into the social sciences [6] and medicine [7, 8]. Some philosophers have argued that knowledge of mechanisms can help solve the problem of applying average medical study results to target populations [9–15]. This is alternatively referred to as the problem of ‘external validity’, ‘generalizability’, and ‘extrapolation’. Following some work in the philosophical literature [10–13], we use the term ‘problem of extrapolation’.

In this paper, we explore how knowledge of underlying mechanisms might solve the problem of extrapolation. We shall argue that apart from a few cases, serious obstacles prevent mechanisms from offering a robust tool to solve the problem. We begin by describing the problem of extrapolation, defining mechanisms, and outlining how knowledge of mechanisms offer a solution. We then describe four often-overlooked problems with using mechanistic knowledge to solve the problem of extrapolation. First, our knowledge of underlying mechanisms is often mistaken or incomplete. Secondly, knowledge of mechanisms often cannot be justifiably extrapolated outside the tightly controlled laboratory situations in which such knowledge is usually produced. Thirdly, mechanisms can behave paradoxically. Finally, using mechanistic knowledge does not overcome what Dan Steel calls ‘the extrapolator’s circle’. It would be a mistake, however, to claim that knowledge of mechanisms never helps mitigate the problem of extrapolation. We provide examples of exceptional cases in which mechanistic knowledge is helpful. We conclude that while mechanistic reasoning can be useful for solving the problem of extrapolation in some cases, one may have to look elsewhere for more robust solutions. Until such solutions are found, one may have to adopt a higher degree of scepticism about the applicability of results from controlled studies to target populations.

## Why it is problematic to apply the results of controlled studies to target populations

Average study results may not apply to individuals or subgroups *within a study*, or to target populations which are sometimes relevantly different from study populations. This problem is commonly discussed in the context of randomized trials, but it also applies to controlled observational studies and, as we shall point out below, results from studies that investigate underlying mechanisms. It is a problem whether the studies are analysed using frequentist or Bayesian methods [16].

Consider the following imaginary example. If half the participants in a trial experienced 100% recovery, and the other half experienced no effect, the average outcome (50% recovery) would not describe what happened to any particular individual in the study. In a real example taken from Peter Rothwell [17], investigators conducting the European Carotid Surgery Trial (ECST) found that carotid endarterectomy appeared to carry an obvious risk (an approximately 0.5% increase in mortality) [18, 19]. However, when Rothwell restricted the analysis to patients with severe carotid stenosis, the intervention was found to be beneficial [17]. This is not a problem with implementing the study results to populations outside the trial; hence, the term ‘external validity’ is misleading. Unless there is no variation, average study results may not even apply to individuals *within* the trial.

In addition, target populations can be different from study populations. Up to 90% of potentially eligible participants are sometimes excluded from trials according to often poorly reported and even haphazard criteria [20–25]. For example, even the most effective antidepressants in adults have doubtful effects in children [26, 27]. In another example taken from John Worrall [28], the drug benoxaprofen (Oraflex™ in the USA and Opren™ in Europe) proved effective in trials in 18–65 year-olds, but killed a significant number of elderly patients when it was introduced into routine practice. The problem that average results do not apply to individuals or subgroups within a trial is exacerbated by the fact that people can change over time. Results from a study that were applicable at time T_1_ might not apply at a different time T_2_.

Besides differences between people in study and target populations, study and target contexts can differ. In a presidential address to the Philosophy of Science Association [15], Nancy Cartwright illustrated this aspect of the problem with the example of the Tamil Nadu Integrated Nutrition Programs (TINP I and TINP II). These programs aimed to improve the nutritional status of preschool children (6–36 months old) and pregnant and nursing women. To achieve the aim, investigators provided a package of services that included nutrition education, primary health care, supplementary on-site feeding of children, education for diarrhoea management, vitamin A, deworming, supplementary feeding of women, and growth monitoring through monthly weighing of all children aged 6–36 months.

TINP’s success was measured by comparing changes within TINP districts with changes in non-TINP districts. Independent surveys showed that severe malnutrition declined by at least 33% among children aged 6–24 months and by 50% among those aged 6–60 months [29, 30]. TINP II was similarly successful, with a more conservative independent estimate of a 44% decline in severe malnutrition over five years [31, 32].

Inspired by TINP, a similar project called BINP (Bangladesh Integrated Nutrition Project) was implemented in Bangladesh. Unfortunately, BINP enjoyed little success: independent agencies reviewed the evidence and found little reason to believe that the project had had any impact [33, 34]. While the relevant biological traits of the study participants in Tamil Nadu and Bangladesh are unlikely to have been very different, the social contexts in Bangladesh were dissimilar in important ways. The first main difference appeared to be ‘leakage’: the food supplied by the project in Bangladesh was often used as substitutes for other family members rather than supplements for mothers and children. Other related reasons were ‘the mother-in-law factor’ and the ‘man shopper’ factor:

The program targeted the mothers of young children. But mothers are frequently not the decision makers … with respect to the health and nutrition of their children. For a start, women do not go to market in rural Bangladesh; it is men who do the shopping. And for women in joint households—meaning they live with their mother-in-law—as a sizeable minority do, then the mother-in-law heads the women’s domain. [35, p. 6]

To recap, the problem of extrapolation is the problem of justifying claims that average study results apply to ‘target populations’. For present purposes, we shall take target populations to be populations other than average study populations. This includes individuals or subgroups within a study, or populations that were not, and perhaps would not have been, included in a study.

There are at least five (non-exclusive) solutions to the problem of extrapolation. One, simple induction might be used. This is a strategy that some medical researchers, including Iain Chalmers and Mark Petticrew, seem to advocate [36]. But the examples above suffice to reject this as a robust strategy. Moreover, even the most vociferous proponents of simple induction would not hold, for example, that the effects of drugs in plants or animals always apply to humans. Even simple induction (in practice) must be justified by *similarity* between study and target populations. But judgments about relevant similarities come from elsewhere, such as arguments that relevant causal mechanisms are shared.

Two, *n*-of-1 trials [37], in which a single patient randomly receives the experimental treatment or the control for alternating time periods, could be used. The problem of extrapolation does not arise in the context of *n*-of-1 trials, because the the trial population is (usually) the target population. However, *n*-of-1 trials are not applicable outside relatively stable chronic ailments.

Three, pragmatic randomized trials that, insofar as possible, mimic target conditions and have few (if any) exclusion criteria [38], could be considered. However, pragmatic trials do not solve the problem that average results are not always good predictors of individual or sub-group responses. Moreover, no matter how inclusive researchers attempt to make a study, there are likely to be unrepresented populations and circumstances, especially if one considers that circumstances and people change over time.

Four, it is arguable that clinical expertise can be used to determine whether trial results are applicable to target populations or individuals within clinical practice. While expertise may always be required to take variations in patients’ values and circumstances and enhancing placebo effects into account [39], it is unclear how expertise alone (without implicit or explicit appeal to empirical studies) is a source of evidence for whether an intervention is likely to produce a putative effect in a study or target population [40].

Five—and this is the potential solution that we shall examine in this paper—it can be argued that mechanistic knowledge can solve the problem of extrapolation.

## Mechanisms, mechanistic reasoning, and black boxes

To understand how knowledge of mechanisms might solve the problem of extrapolation, we must explain mechanisms, mechanistic reasoning, and evidence from controlled clinical studies.

Philosophers have characterized ‘mechanisms’ in many ways, including the following:

A mechanism is a structure performing a function in virtue of its component parts, component operations, and their organization. The orchestrated functioning of the mechanism is responsible for one or more phenomena. [5, p. 423]

A mechanism underlying a behavior is a complex system which produces that behavior by the interaction of a number of parts according to direct causal laws. [2, p. 52]

Mechanisms are entities and activities organized such that they are productive of regular changes from start or set-up to finish or termination conditions. [1, p. 3]

A nomological machine is a stable enough arrangement of components whose features acting in consort give rise to (relatively) stable input/output relations. [41, p. 8]^1^

There are others besides [42].

**Fig. 1:**
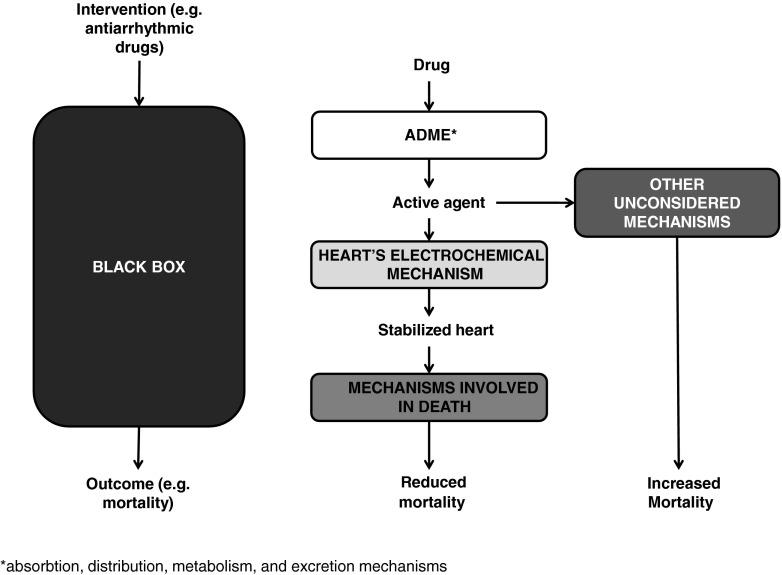
Controlled clinical study and mechanistic reasoning: the example of antiarrhythmic drugs

It is beyond our scope to discuss the differences between these characterizations or their similarities [12, 43], and we contend that our argument applies no matter which of the above characterizations one prefers. Our interest here is epistemological: how can knowledge of mechanisms help us predict whether study results can be successfully implemented? Such alleged knowledge must rest on claims about a mechanism’s action. Whether the mechanism’s action is called ‘orchestrated functioning responsible for one or more phenomena’ (William Bechtel and Adele Abrahamsen) [5], ‘behavior production’ (Stuart Glennan) [44], ‘regular change production’ (Peter Machamer, Lindley Darden, Carl Craver) [1], or ‘action’ (Cartwright) [45] is immaterial to our purpose. Regardless of how they are characterized, mechanisms must have some action if they are to be used to support claims that an intervention produces some effect. Following previous work, we define ‘mechanistic reasoning’ as an inference about an intervention’s clinical effect from alleged knowledge of relevant mechanisms and how they relate to one another [46, 47]. By contrast, a controlled trial is a ‘black box’ as far as the inner workings of an intervention are concerned (see Fig. 1, left-hand side).

Typically, in clinical medicine, more than one mechanism is involved in producing a patient-relevant effect (see Fig. 1, middle). Consider the example of mechanistic reasoning that was used to support claims that antiarrhythmic drugs reduce mortality in certain patients. Several mechanisms (swallowing, gastric emptying, metabolism, circulatory, and binding mechanisms) might be involved in getting the drug to its pharmacological targets. These mechanisms are often well understood and are referred to as ADME (mechanisms for absorption, distribution, metabolism, and excretion). Having reached their cellular targets, antiarrhythmic drugs were believed to reduce the frequency of ventricular extra beats by modifying the heart’s electrochemical mechanism. Finally, a reduction in ventricular extra beats should (allegedly) reduce the risk of sudden death, presumably by modifying the circulatory mechanism (by reducing the risks associated with insufficient blood flow to vital organs).

It is generally possible to describe the mechanistic chain or web at different levels. In the antiarrhythmic drug example, we might have categorized the component *mechanisms* (ADME, actions on the heart, etc.) as *parts* (or entities or components) of a larger mechanism (the human body). Likewise, we might have chosen the molecular or even subatomic level. We chose to refer to the ADME and heart mechanisms in the antiarrhythmic drug example because they map most directly on to the language used by medical researchers.

In any case, the choice of descriptive level [48], or indeed, how one characterizes mechanisms, does not affect our arguments. The essential feature of mechanistic reasoning is that it involves an inferential chain (or web) linking the intervention with a clinically relevant outcome via (productive!) mechanisms. If the productive capacities of the mechanisms linking the intervention with the clinical effect can be established, then we have good evidence in the form of what we call ‘mechanistic reasoning’ no matter how the productive ability of the mechanisms is characterized.

## How knowledge of mechanisms allegedly solves the problem of extrapolation

Philosophers of science often disagree with evidence-based medicine (EBM) proponents about the role of mechanisms for supporting claims about efficacy, but they seem to agree about the role of mechanisms when it comes to extrapolation [39]. Some influential proponents of EBM have stated, for example, that:

A sound understanding of pathophysiology is necessary to interpret and apply the results of clinical research. For instance, most patients to whom we would like to generalize the results of randomized trials would, for one reason or another, not have been enrolled in the most relevant study. The patient may be too old, be too sick, have other underlying illnesses, or be uncooperative. Understanding the underlying pathophysiology allows the clinician to better judge whether the results are applicable to the patient at hand. [49, p. 2423]

This advice continues in three editions of an EBM textbook [50–52], and critics of EBM also share this view [53]. To be sure, the term used by some EBM proponents (‘pathophysiologic rationale’) appears to be different from our ‘mechanistic reasoning’. At the same time, pathophysiology involves the study of how bodily processes behave in normal and abnormal circumstances [54], and ‘rationale’ is a synonym of ‘reasoning’ [54]. Hence, we take ‘pathophysiologic rationale’ to mean (roughly) the same as ‘mechanistic reasoning’.

By way of support for the EBM view, Gordon Guyatt and Paul Glasziou (in conversation) have offered the following illustration. A trial might exclude everyone over the age of 60. They claim that mechanistic considerations support the view that the intervention is likely to work for a 61 year-old but may not work for a 90 year-old. Presumably, they take it that the success of the intervention depends on the operation of pathophysiologic mechanisms that change only slowly beyond 60 and so would not have changed substantially in most 61 year-olds but would be highly likely to have changed by the time they are 90.

In a growing body of literature that began with discussions of the applicability of results from animal studies to humans, philosophers of science have taken what may be interpreted as a position very similar to that of many EBM proponents. These philosophers of science have argued that knowledge of mechanisms can justify implementing average study results to target populations by *analogy* [9–15]. On this view, extrapolation is justified insofar as the relevant mechanisms—and hence the mechanistic reasoning linking the intervention and outcome—are shared in the study and target populations.

Dan Steel is the philosopher of science who has written most extensively on the subject and he correctly points out that this simple mechanistic solution to the problem fails because of the ‘extrapolator’s circle’ [12, 13]. In order to determine whether the mechanism in the target is sufficiently similar to the mechanism in the study population to justify extrapolation, one must know how relevant mechanisms in the target behave. But, Steel argues, if one had knowledge of mechanisms in the target population, then one would have strong mechanistic reasoning supporting the claim that the intervention caused the outcome in the target population. This would make the initial study (in the model) redundant. In Steel’s words, ‘it needs to be explained how we could know that the model and the target are similar in causally relevant respects without already knowing the causal relationship in the target’ [12, p. 78].

To escape from this circle, Steel offers a more sophisticated account of how mechanistic knowledge might help us justify implementing study results, namely, *comparative process tracing*. Comparative process tracing involves two steps:

1. ‘Learn the mechanism in the model organism, by means of process tracing or other experimental means’ [12, p. 89]. ‘Process tracing’ involves a step-by-step reconstruction of the path connecting an end-point (an initial cause or a final effect) with other elements of the mechanism via intermediate nodes.
2. ‘Second, compare stages of the mechanism in the model organism with that of the target organism in which the two are most likely to differ significantly’ [12, p. 89].

A key feature of Steel’s account is that one need not know everything about the mechanisms in the target, but only the relevant parts of the mechanism, namely, those that are likely to differ significantly. Often, the needed points of comparison can be limited to stages of the mechanism close to the endpoint—the reasoning being that differences upstream matter only if they generate differences further downstream. This significantly reduces the number of points in the mechanism that need to be compared. Hence, one need not know everything about the mechanism in the target in advance, and the extrapolator’s circle is allegedly avoided.

In spite of its intuitive appeal, mechanistic reasoning, even in Steel’s more sophisticated account, is plagued by several problems that make it unsuitable as a robust solution to the problem of extrapolation.

## Problems with mechanistic knowledge for solving the problem of extrapolation

### (Epistemological) problems with identifying relevant mechanisms

Mechanistic reasoning will be useful only insofar as relevant mechanisms are correctly identified and understood. But correct identification of all relevant mechanisms in any population is far more difficult than is often presumed. For example, a plausible (but incorrect) mechanism for blood creation led to various erroneous diagnoses and treatments such as bloodletting. Even if some mechanisms are correctly identified, other mechanisms (or features of mechanisms) are often missed. This can lead to mistaken predictions about efficacy, and in the case of extrapolation, the mistaken claim that mechanistic reasoning in study and target mechanisms are shared. To see how even apparently sensible mechanisms can lead to mistaken predictions, recall that mechanistic reasoning supported the view that anti-arrhythmic drugs would reduce mortality. However, a subsequent randomized trial suggested that the reasoning was mistaken. In the Cardiac Arrhythmia Suppression Trial (CAST), 1,827 patients were randomized after myocardial infarction to receive antiarrhythmic drugs (encainide, flecainide, or moricizine) or placebo. Ten months later the antiarrhythmic drugs were discontinued because of excess mortality: 4.5% of those who took either encainide or flecainide had died of arrhythmias or cardiac arrest, while only 1.2% of those who took placebo had died for similar reasons [55]. The experimental drugs also accounted for 4.7% greater all-cause mortality (see Fig. 1, right hand side). The drugs activated an unsuspected mechanism that increased mortality.

Even in areas that are very well understood, such as the cholesterol pathway, drugs can activate unexpected mechanisms, with dramatic consequences [56]. Thalidomide, for example, was introduced to relieve morning sickness but was later found to cause severe birth defects. Surprising side effects can also be positive. Sildenafil was originally designed to treat angina, but in the first clinical trials, it revealed the surprising effect of producing penile erection; it was subsequently marketed as *Viagra™* and became a huge commercial success.

Steel would presumably reject the claim that all relevant mechanisms need to be identified, because it is often allegedly sufficient to identify downstream stages (‘bottlenecks’) through which the eventual clinical outcome must be produced. However, this raises the issue of how researchers know that they have identified the bottlenecks correctly, and whether they are sure they have not missed some additional mechanisms activated by the intervention but bypassing the bottleneck. The antiarrhythmic drug example and many others [57, 58] suggest that our knowledge of mechanisms is often lacking. Indeed some have argued that medicine did more harm than good until quite recently, precisely because of reliance on faulty or incomplete knowledge of mechanisms [59]. Steel fails to acknowledge this literature and hence leaves us wondering how mechanistic reasoning that is grounded in sufficient knowledge of mechanisms can be distinguished from mechanistic reasoning based on incomplete or mistaken alleged knowledge of mechanisms.

### Why studies of mechanisms suffer from problems of generalizability

The functioning of most mechanisms is discovered in tightly controlled laboratory experiments that expressly exclude as many potentially interfering variables as possible. Why would effects discovered in tightly controlled laboratory circumstances generalize more readily than effects discovered in controlled clinical studies? If they do not, then any knowledge about the mechanisms gained in these controlled settings is less likely to be shared by ‘real world’ populations. For example, St. John’s wort has been shown in laboratory settings to induce the activity of cytochrome P450 (CYP) isoenzymes, which are extensively involved in metabolizing about 50% of known drugs [60], including many steroids. However, a clinical study suggested St. John’s wort did not reduce the concentrations of androgenic steroids [61], presumably because of some compensatory mechanism. In this example the behaviour of a mechanism in the laboratory was not reproducible in a real clinical setting. Knowledge of mechanisms gained in these tightly controlled contexts may differ relevantly from mechanisms in both trial and target populations and therefore cannot straightforwardly be used to justify claims about similarity between trial and target populations.

### The unwarranted ontological assumption that mechanisms are productive of regular relationships between inputs and outputs

Claude Bernard, perhaps the grandfather of contemporary mechanistic reasoning in medicine, believed that mechanisms were productive of stable deterministic laws that precluded the need for any further ‘empirical’ evidence (for example, from controlled studies). He stated for example that:

Now that the cause of the itch is known and experimentally determined, it has all become scientific, and empiricism has disappeared. We know the tick, and by it we explain the transmission of the itch, the skin changes and the cure, which is only the tick’s death through appropriate application of toxic agents.… We cure it *always* without any exception, when we place ourselves in the known experimental conditions for reaching this goal. [62, p. 214]

While few today believe that more than a handful of diseases (if any!) are cured ‘always and without exception’ [63]—and indeed Claude Bernard himself advocated clinical trials when mechanisms were unknown [64]—the belief that mechanisms produce stable relationships is widely held among mechanist philosophers of science. Consider other excerpts from the recent literature.

[Mechanisms are] entities and activities organized such that they are productive of *regular* changes from start or set-up to finish or termination conditions. [1, p. 3 emphasis added]

The existence of a mechanism provides evidence of the *stability* of a causal relationship. If we can single out a plausible mechanism, then that mechanism is likely to occur in a range of individuals, making the causal relation stable over a variety of populations. [7, p. 159, emphasis added]

Nomological machines [mechanisms] generate causal laws between inputs and *predictable* outputs. [65, p. 156, emphasis added]

The belief that mechanisms are productive of stable relationships might be borrowed from mechanics, where, if the quantum level is ignored, there *are* many mechanisms productive of stable input-output relationships. For instance, Cartwright cites the example of a toaster’s mechanism [41]. But mechanisms in the human body and social world, especially those that are pertinent to clinically relevant outcomes, are generally far more complex than toasters and other mechanical machines. Besides the epistemological problems with discovering any assumed regularity (such as extreme sensitivity to initial conditions and complex interactions), mechanisms themselves might not behave regularly at all [66].

Mechanisms’ irregular behaviour is perhaps best exemplified by paradoxical reactions. Smith et al. have listed many drugs that sometimes worsen the condition for which they are indicated [67]. To name a few, antiepileptic drugs can both prevent and cause seizures [68, 69], antidepressants can both ameliorate and worsen depressive symptoms [70, 71], and antiarrhythmic drugs can cause arrhythmias [72]. Even the same molecule can initiate different mechanisms depending on its environment within the body.

If mechanisms can have paradoxical and unanticipated effects, then even if it is established that some mechanisms in the study and target populations are shared, one cannot know whether they will behave the same way in different populations. A supporter of mechanistic reasoning might, of course, claim that the paradoxical behaviour of the mechanism is simply a sign that some other mechanism (or feature of the mechanism) that can explain the paradox is yet to be identified. But this objection seems to rely on a determinist metaphysics that requires independent arguments.

To recap, mechanistic reasoning as a strategy for solving the problem of extrapolation faces several hitherto unmet challenges. We will now argue in more detail that neither Steel’s comparative process tracing nor Cartwright’s account overcomes the problems we have pointed out above.

### How Daniel Steel’s comparative process tracing does not avoid the extrapolator’s circle

Recall Steel’s argument that comparative process tracing is a mechanist solution to the problem of extrapolation that does not fall into the extrapolator’s circle. Comparative process tracing relies on ‘[j]udgments about where significant differences are and are not likely to occur … based on inductive inferences concerning known similarities in related mechanisms in a class of organisms, and on the impact those differences make’ [12, p. 89]. In short, Steel divides parts of the mechanisms (in both the model and target) into two categories: those that are known (or suspected) to be similar, and those for which significant differences are likely.

Consider the single example (the carcinogenic effects of the aflatoxin AFB_1_) that Steel offers in support of his thesis:

It was found that AFB_1_, the most common aflatoxin, was converted to the same phase I metabolite across [human and rodent] groups…. Given the sharp differences in carcinogenic effects of AFB_1_ in rats and mice, it was of obvious interest to inquire which of these two animal models was a better guide for humans. It was found that although the phase I metabolism of AFB_1_ proceeded similarly among mice, rats, and humans (and in fact at a higher rate in mice), the phase II metabolism among mice was extremely effective in detoxifying AFB_1_ but not among rats or humans…. Furthermore, this metabolite bound to DNA in rat liver cells in vivo at sites at which the nucleotide base guanine was present to form complexes called DNA adducts…. It was further found that such cells suffered unusually frequent mutations in which guanine-cytosine base pairs were replaced with adenine-thymine pairs, a mutagenic effect found in vivo among rats and in vitro among cells of a variety of origins, including bacteria and human [cells]…. In addition, guanine-cytosine to adenine-thymine mutations were found in activated oncogenes present in rats exposed to AFB_1_ but were absent in the controls…. Thus, comparative process tracing yielded the conclusion that the rat was a better model than the mouse. [12, p. 91]

This example—and comparative tracing in general—does not support the view that comparative process tracing escapes the extrapolator’s circle. First, consider the parts of the mechanism that are allegedly known to be similar (phase I metabolism in the aflatoxin example). In order to establish that phase I metabolism was similar across groups, Steel cites a study involving humans as well as rodents [73]. But once the study in humans is available, the rodent study becomes redundant and Steel faces the extrapolator’s circle.

This leaves the parts of the mechanism that are suspected to differ between the target and model. In Steel’s example, the phase II similarity between rats and humans (and the difference between mice and humans) was likely to differ but the similarity was allegedly established by a study in humans. This makes the study in rats redundant. Moreover, the study Steel cites in support of the view that rats are better models than mice [74] does not involve clinical outcomes, but merely in vitro studies of human blood samples.

In short, for both categories of comparisons (those in which model and target mechanisms are likely to be similar and those in which model and target mechanisms are likely to be different), the study of the model is redundant and comparative process tracing does not escape the extrapolator’s circle.

Steel’s claim that all similarities and differences need not be known provided that ‘bottlenecks through which any influence on the outcome must be transmitted’ can be found [12, p. 90] does not save his argument. Besides the problem of correctly identifying bottlenecks (see above), this potential reply slips back into the extrapolator’s circle: if one knows where the bottlenecks are in the target, then the knowledge of the mechanism in the study population (at least upstream from the bottleneck) becomes redundant. As for the mechanisms downstream from a bottleneck, either they are known to be similar or known to be different. In each case, studies of the target are required to establish the similarity or difference and the extrapolator’s circle re-emerges.

### Cartwright’s example fails to support the view that mechanisms can solve the problem of extrapolation

With the common problems with mechanistic reasoning in mind, we now revisit Cartwright’s TINP example to show why it does not support using mechanistic reasoning to solve the problem of extrapolation. The ‘man shopper’ and ‘mother-in-law’ factors in the BINP study upset the mechanism that was effective in the TINP study by preventing delivery of the food to the children’s stomachs. This post-hoc explanation *might* have informed policy makers how to modify BINP and prevented its failure. However, knowledge of the different mechanisms might have produced harm. Imagine that the World Bank hired consultants who correctly identified the problems. The consultants might reasonably propose to deliver the food directly to the mother, and not allow the men or mothers-in-law to lay their hands on it (or alternatively ‘educate’ the mothers-in-law and men). But such a plan could easily backfire: the mothers-in-law and fathers could feel resentful and become abusive towards mothers and children. In this imaginary—but sadly by no means implausible—example, appeal to mechanisms when extrapolated would lead to harm. The cause of the failure would have been the inability to identify all relevant mechanisms activated by the modified intervention. Ironically, if all relevant mechanisms had been identified, then investigators would have fallen into the extrapolator’s circle!

To recap, mechanistic reasoning provides *prima facie* promise for solving the problem of extrapolation, but several obstacles stand in the way of its providing an actual solution. First, it is rarely possible to identify all relevant mechanisms. Second, studies of mechanisms themselves (whether in animals or humans) suffer from their own problems of ‘external validity’. Third, mechanisms can behave paradoxically. Steel’s comparative process tracing fails to solve the problem, and contrary to what Cartwright asserts, appealing to knowledge of mechanisms to solve the problem of extrapolation can harm rather than help. At the same time, there are some well-defined cases in which mechanistic knowledge can provide a reliable solution to the problem of extrapolation.

## When mechanistic knowledge can help justify applying average study results to target populations

The limits to our knowledge of mechanisms listed above must temper our confidence in all mechanistic reasoning, whether it is used to establish efficacy [46, 47] or to solve the problem of extrapolation. However, some claims about mechanisms are based on stronger evidence than others [46], and in these cases mechanistic reasoning can be used to justify extrapolation. For example, the proximate causes of stroke have been known for centuries [75, 76]. A burst artery in the brain causes a haemorrhagic stroke, while an ischemic stroke is caused by a blockage of an artery that supplies blood to the brain, by either thrombosis or embolism. Aspirin benefits patients who have had an ischemic stroke, but may harm those who have had a haemorrhagic stroke. The cause of the stroke (identification of the mechanism that has been disturbed) can be discovered by a CT scan. In this case, extrapolation of studies (of the treatments for ischemic or haemorrhagic stroke) to individual patients uses mechanistic reasoning to classify patients into groups that are likely to benefit or not from an intervention.

To cite another example of how understanding mechanisms can reduce harmful extrapolation, recall from earlier that the drug benoxaprofen (Oraflex™ in the USA and Opren™ in Europe) proved effective in clinical trials, but killed some elderly patients when it was used in routine practice [77]. This was due to altered pharmacokinetics in the elderly patients, which should have been suspected, based on what is known about the physiology and pathology of ageing; frail elderly subjects have reduced liver function and benoxaprofen is metabolized in the liver. There are other well-known examples of effect modification by age, including antihypertensive drug treatments, which reduce total mortality in middle-aged patients but may not do so in elderly ones [78], and reducing dosages of growth hormone for adults with growth hormone deficiency [79, 80].

## Conclusion

The problem of extrapolation is real, and simple induction fails in many important cases. In this paper we have evaluated mechanistic knowledge as a potential solution to the problem and concluded it is rarely successful. We have illustrated four often overlooked problems with using mechanistic knowledge for solving the problem of applicability: current knowledge of mechanisms is often mistaken, the mechanistic knowledge itself can lack external validity, mechanisms can behave paradoxically, and the mechanist solution does not overcome the problem of the extrapolator’s circle. Where these problems have been addressed, knowledge of mechanisms can mitigate the problem of extrapolation, often by sounding a bell of caution when implementing study results to target populations whose mechanisms are known to differ significantly.

When mechanistic understanding is lacking, how might extrapolation of study results to target populations be justified? Certainly more systematic investigations of the various potential solutions described in this paper (pragmatic trials, *n*-of-1 trials, and clinical expertise) are warranted. Or, an intervention that shows promise in a trial could be rolled out to target populations slowly, and modified according to what is systematically observed. A possibility that has been implied throughout this paper is that we have to learn to live with a much higher degree of uncertainty and scepticism about the effects of many medical interventions, even those whose effects have been established in well-controlled population studies.

## Footnotes

1 Cartwright (personal communication) claims that her ‘nomological machines’ fall into the general category of ‘mechanisms’ as described by the other authors cited above.
